# Cyclical vomiting syndrome secondary to a Chiari I malformation—a case report

**DOI:** 10.1007/s00381-017-3589-5

**Published:** 2017-09-11

**Authors:** William L White, Veejay Bagga, David I Campbell, Anthony R Hart, Shungu Ushewokunze

**Affiliations:** 1 The Medical School, University of Sheffield, Beech Hill Road, Sheffield, S10 2RX UK; 2 Sheffield Children’s Hospital NHS Foundation Trust, Western Bank, Sheffield, S10 2TH UK; 3 Department of Neurosurgery, Sheffield Children’s Hospital NHS Foundation Trust, Sheffield, UK; 4 Department of Gastroenterology, Sheffield Children’s Hospital NHS Foundation Trust, Sheffield, UK; 5 Department of Neurology, Sheffield Children’s Hospital NHS Foundation Trust, Sheffield, UK

**Keywords:** Chiari malformation type I, Cyclical vomiting syndrome, Craniocervical decompression

## Abstract

**Background:**

Cyclical vomiting syndrome is a disorder characterised by recurrent episodes of profuse vomiting. There are no cases in the literature on the management of children with presenting with cyclical vomiting syndrome and a Chiari malformation type I.

**Discussion:**

We report the case of a 12-year-old child diagnosed with cyclical vomiting syndrome and a Chiari malformation type I. The patient received symptomatic relief following a craniocervical decompression.

## Introduction

Cyclical vomiting syndrome (CVS) is a disorder characterised by recurrent episodes of profuse vomiting separated by symptom-free periods [1, 2]. The aetiology and pathogenesis of CVS is unknown [1] but may overlap with migraine because of responsiveness to anti-migraine drugs and the frequent presence of a migraine-type prodrome. The diagnosis of CVS is one of exclusion after clinicians have excluded gastrointestinal, metabolic and central nervous system causes of vomiting.

Chiari malformation type I (CM-I) is a situation where the cerebellar tonsil protrudes below the foramen magnum and into the spinal canal, affecting an estimated 0.5–3.5% of the general population [3]. Patients with CM-I may be asymptomatic or may present with symptoms such as Valsalva headaches, vomiting or symptoms of raised intracranial pressure (ICP) secondary to hydrocephalus [3].

To the authors’ knowledge, only one previous report demonstrates a link between a CM-I and CVS [1]. Here, we report a case of a 12-year-old boy with CVS and a CM-I who underwent a craniocervical decompression after other known causes of his vomiting were excluded.

## Case presentation

A 12-year-old boy presented with a 12-month history of recurrent episodes of vomiting. The vomiting occurred daily, up to 32 times a day, and was associated with a prodrome of “stomach churning” immediately before vomiting. The episodes progressively worsened, preventing him from attending school regularly and engaging in physical activities. On examination, there were no neurological abnormalities. Extensive investigations including upper gastrointestinal endoscopy with histological examination of the upper GI tract, pH studies, coeliac serology, metabolic and toxicology screens were negative. An MRI scan of the abdomen and an electroencephalogram also detected no abnormalities. He was subsequently diagnosed with CVS.

**Fig. 1:**
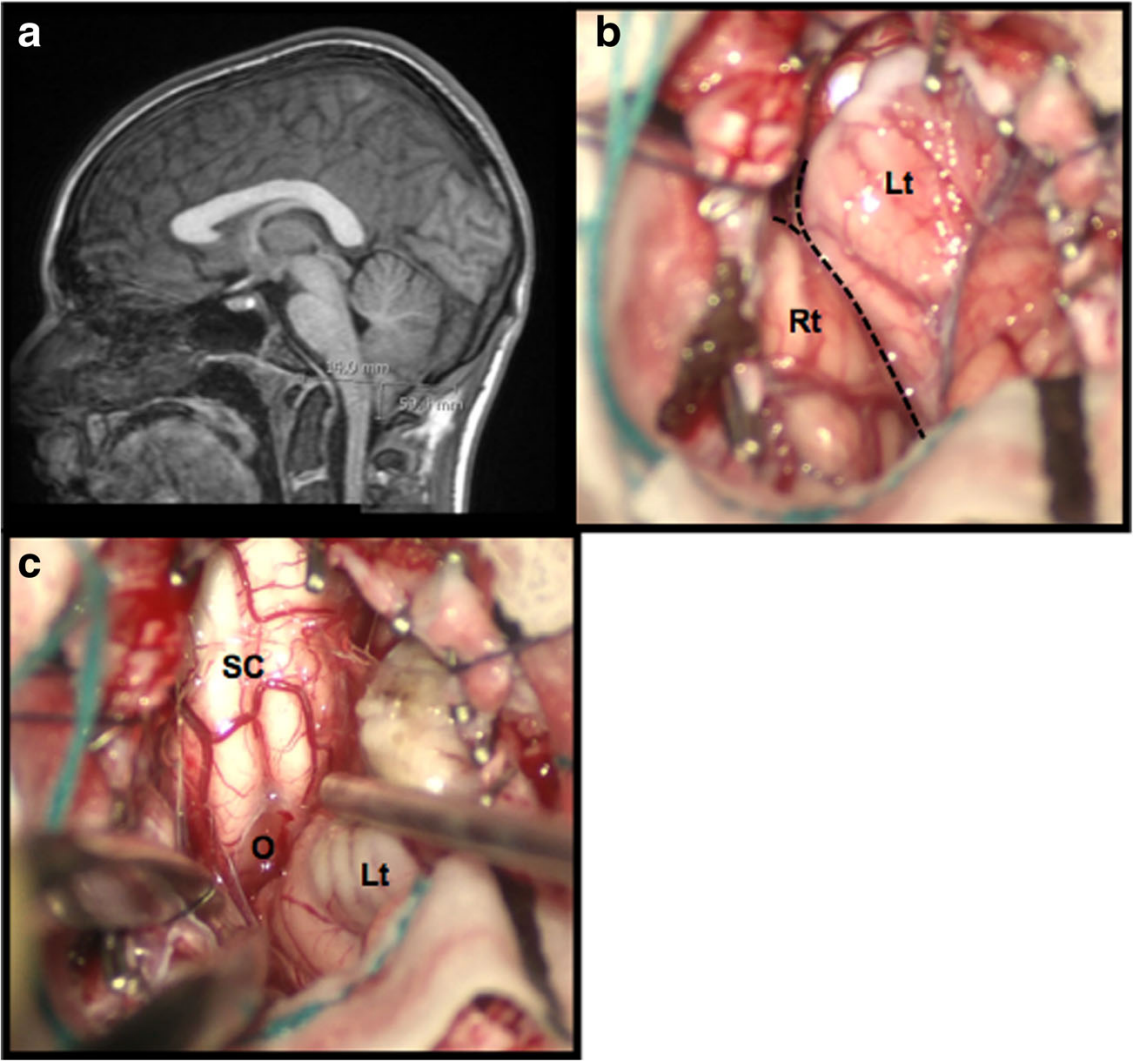
**a** Sagittal MRI showing caudal descent of the cerebellar tonsils. **b**–**c** Intra-operative images following suboccipital craniotomy and removal of the posterior arch of C1. Large cerebellar tonsils can be seen (separated by dashed line). The left tonsil - Lt is causing lateral displacement of the right tonsil (Rt) (**b**). Following coagulation, the tonsils have been reduced in size allowing exposure of the dorsal spinal cord (SC) and the obex of the 4th ventricle (O) (**c**)

For completion, a magnetic resonance imaging (MRI) scan of the brain and spine was performed, which revealed a CM-I (Fig. 1a). He was discussed in the neurosciences multidisciplinary team meeting and the CM-I was considered an incidental finding. A trial of propranolol did not control his symptoms and was discontinued owing to hypotension. ICP monitoring was organised for 48 h, which did not show any rises in ICP. As the patient’s symptoms were progressing, the role of surgery was discussed with the family as a last option and a craniocervical decompression was performed.

Intra-operatively, the cerebellar tonsils were found to be very large with the left tonsil causing lateral displacement of the right tonsil and caudal displacement below the level C1 (Fig. 1). The cerebellar tonsils were causing direct pressure on the underlying cervicomedullary junction and were reduced by coagulation, allowing free flow of cerebrospinal fluid. A duraplasty using a pericranial graft was performed. He was discharged after 5 days. During his post-operative inpatient stay, he did not have any episodes of vomiting; however, he did require a small amount of anti-emetics immediately post-operatively. There were no complications associated with the surgery. At 20-month follow-up, he reported no episodes of vomiting since surgery, has returned to full-time education and was discharged from follow-up.

## Discussion

CVS is characterised by intense episodes of recurrent vomiting occurring at least four times per hour for at least an hour, followed by symptom-free periods. It is often misdiagnosed [1] as gastroesophageal reflux or an intercurrent viral infection. The pathogenesis and aetiology of CVS is currently unknown, but multiple hypothesises have been proposed including migraine-related mechanisms and mitochondrial DNA mutations, along with neuroendocrine and autonomic dysfunction [1]. The literature also describes an association between CVS and CM-I, although does not describe the management of patients with a CM-I presenting with CVS [1]. Furthermore, there is no previous evidence to support the role of surgical decompression in children with CVS. We are the first to demonstrate a resolution of symptoms following a craniocervical decompression in a patient with CVS and a CM-I.

The pathogenesis in our patient is most likely due to direct pressure on the brainstem. Vomiting is a complex physiological reflex controlled by two centres in the brainstem. The vomiting centre is located in the dorsolateral reticular formation in the medulla. The chemoreceptor trigger zone is located in the area postrema of the medulla and the lateral walls of the fourth ventricle outside the blood brain barrier [4]. The vomiting centre is particularly sensitive to changes in ICP. In our patient, the 48 h of ICP monitoring did not show any evidence of raised ICP. At surgery, it was evident that the left cerebellar tonsil was causing direct pressure on the medulla. In view of the good response to surgery, it is reasonable to deduce that the cause of the recurrent vomiting episodes was due to a direct effect of pressure on the medulla.

The pathogenesis of CVS is likely to be influenced by multiple factors, including raised ICP. Therefore, before a diagnosis of CVS is made, patients must have a full work up to exclude all possible organic causes. If no obvious cause is identified, an MRI of the brain should be performed as causes of raised ICP and direct pressure on the brainstem need to be excluded [2]. If a CM-I is diagnosed and there is failure of medical treatment for CVS, craniocervical decompressive surgery should be considered.
